# Dynamic digital radiography is a valid and reliable alternative to 3 Dimensional registration for scapulohumeral rhythm analysis

**DOI:** 10.1016/j.jseint.2026.101697

**Published:** 2026-03-16

**Authors:** Sameer R. Khawaja, Jaden C. Hardrick, Zaamin B. Hussain, Itaru Kawashima, Joanne Y. Zhou, Hayden L. Cooke, Krishna N. Chopra, Scott A. Banks, Michael B. Gottschalk, Thomas W. Wright, Eric R. Wagner

**Affiliations:** a Department of Orthopaedic Surgery, Baylor College of Medicine, Houston, TX, USA; b Department of Orthopaedic Surgery, Emory University School of Medicine, Atlanta, GA, USA; c Department of Orthopaedic Surgery and Sports Medicine, University of Florida, Gainesville, FL, USA

**Keywords:** Dynamic digital radiography, Scapulohumeral rhythm, Glenohumeral, Scapulothoracic, Shoulder biomechanics, Kinematics

## Abstract

**Background:**

Scapulohumeral rhythm (SHR) is a key metric for evaluating shoulder joint function. Current established methods of SHR analysis involve 3-dimensional (3D) to 2-dimensional (2D) registration, which requires significantly greater resources to generate dynamic positioning data of the humerus and scapula. Dynamic digital radiography (DDR) captures pulsed low-dose radiographs in the plane of the scapular elevation to create a cine loop for analysis. This study aimed to establish the most reliable measurement technique by identifying bony landmarks that minimize measurement error and validate manual SHR measurements on DDR against an established 3D to 2D registration technique.

**Methods:**

DDRs performed on 22 reverse shoulder arthroplasty shoulders at least 6 months post-operatively were obtained. Manual measurements, including glenohumeral and scapulothoracic angles, were performed on DDRs by 2 authors every 10° of motion from rest to 120° of shoulder elevation. The scapulothoracic angles were measured using either the lateral or medial border of the scapula or scapula spine. Manual measurements were used to calculate SHR across the total range of motion and between various intervals.

**Results:**

SHR values were also calculated from a 3D to 2D registration model. Interclass correlations were statistically significant for glenohumeral angles and scapulothoracic angles using the lateral border – 0.989 (*P* < .001) and 0.955 (*P* < .001), respectively. Intraclass correlations were statistically significant for the glenohumeral angles (0.999 [*P* < .001]) and all scapulothoracic angles using the lateral border, medial border, and scapular spine – 0.999 (*P* < .001), 0.963 (*P* < .001), and 0.989 (*P* < .001), respectively. Paired *t*-tests revealed no significant differences in SHR between manual and 3D to 2D registration measurements across all intervals of shoulder elevation.

**Discussion:**

SHR measurements on DDR images are a simple and valid technique that can be readily incorporated into clinical workflows, where advanced 3D modeling may be impractical due to resource constraints. DDR provides a novel imaging modality to analyze in vivo shoulder biomechanics with comparable accuracy and validity to that of resource-restricted and time-consuming 3D imaging modalities.

Shoulder kinematics involves the complex interactions between the scapula and humerus, with Codman et al first coining this relationship as scapulohumeral rhythm (SHR)—the ratio of glenohumeral (GH) elevation to scapulothoracic (ST) upward rotation.^7^ Though traditionally described as a 2:1 ratio,^18^ recent literature has reported the SHR of normal physiologic shoulders to vary from 2.2:1 to 2.7:1 based on measurement technique,^4^^,^^13^ measurement error,^48^ and intersubject variation,^23^^,^^37^ and even subtle deviations from normal SHR may indicate pathologic motion. Larger SHR ratios (greater than 3.0:1) indicate a higher GH component of shoulder motion relative to ST contribution, whereas smaller SHR ratios (less than 2.0:1) indicate an abnormally increased, and often compensatory, ST contribution.^48^ Accurately assessing SHR and scapular positioning motion is critical in the diagnosis and management of complex shoulder problems. For example, increasing SHR has been associated with improved patient-reported outcome measures after shoulder arthroplasty.^20^ Indeed, its role may be particularly important in shoulder arthroplasty outcomes but is not routinely considered during pre-operative planning or post-operative analyses.^40^

Highly accurate approaches for quantifying in vivo scapular motion include biplane fluoroscopy^13^ and 3-dimensional (3D) to 2-dimensional (2D) registration techniques, which allow precise analyses of shoulder kinematics across several planes of shoulder motion.^6^^,^^36^^,^^44^ Although highly accurate, both biplane fluoroscopy and 3D registration techniques are not conducive to a clinical practice, as they are high radiation exposure techniques, resource intensive requiring a specialized laboratory, and time-consuming with limited automation and scaling capabilities. Additionally, dynamically tracking scapular upward rotation, posterior tilt, and external rotation remains difficult as traditional methods of measuring scapular motion, such as goniometry and palpation, frequently lack accuracy and reliability.^31^^,^^42^ Cadaveric models have been used for research purposes but are unable to replicate in vivo kinematic tension, muscle activation forces, and joint load sharing.

Dynamic digital radiography (DDR) is a novel imaging technique that takes low-dose pulsed radiographs during joint motion to produce dynamic radiographic sequences. Compared to traditional static and fluoroscopic radiographs, DDR offers a more detailed understanding of an individual's shoulder anatomy and kinematics as it relates to dynamic function, with only minimally increased radiation exposure.^30^^,^^41^^,^^46^^,^^48^ In-office DDR provides an efficient and reproducible method to quantify complex in vivo shoulder biomechanics such as SHR without significantly impacting clinical workflow.^5^^,^^48^ However, its effectiveness at objectively assessing SHR and upward rotation of the scapula depends on the identification of non-occluded, straight-segment bony landmarks. With respect to the DDR-based assessment of SHR, the utility of specific scapular landmarks remains to be established.

This study sought to evaluate DDR's reliability and accuracy in assessing SHR by identifying bony landmarks that minimize SHR measurement error. We also aimed to compare it with an established 3D to 2D registration techniques. We propose that the medial cortex of the humerus and lateral border of the scapula are the most reliable bony landmarks for measuring and calculating SHR. Additionally, we hypothesized that SHR values calculated from DDR would be statistically comparable to those obtained from the 3D to 2D registration technique.

## Methods

### Patient inclusion

Institutional review board approval was received prior to performing this retrospective analysis. Patients with reverse shoulder arthroplasty (rTSA) were chosen for this validation study as a well-fixed baseplate into the scapula represents a radiopaque intraosseous marker, whose axes have been previously reported and its change in size and shape can be used for a surrogate for scapula rotation.^20^^,^^22^ Patients who underwent rTSA for rotator cuff tear arthropathy, osteoarthritis, or massive irreparable rotator cuff tear between the years of 2020 and 2023 by a fellowship-trained shoulder surgeon at a single institution were included. Patients must have obtained DDR images at least 6 months post-operatively, as well as computed tomography (CT) images before and at least 3 months after surgery. Six post-operative months were used as the threshold for DDR based on a previous study reporting no significant difference in shoulder kinematics during scapular plane elevation between 6 months and 1 or 2 years after surgery.^36^ Pre- and post-operative CT images were required for the 3D to 2D model-image registration software. Shoulders were excluded for incomplete or unusable data points and the diagnoses of tumors, fractures, post-traumatic or fracture sequelae.

### Dynamic digital radiography acquisition

**Figure 1:**
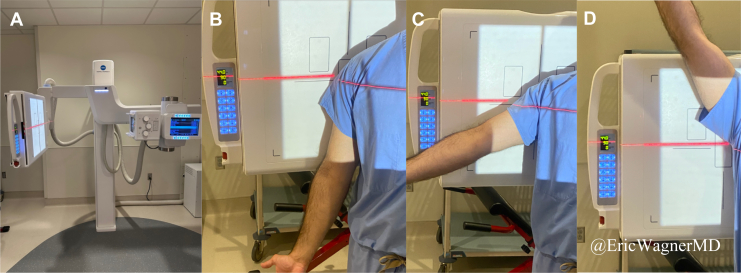
Konica Minolta dynamic digital radiography (DDR) machine (**A**). Patients are positioned in a Grashey view with their posterior shoulder and scapula contacting the back board of the DDR machine. Patients are instructed to stand upright, stationary, avoid axial rotation, and how to perform the motion including standardizing for forearm supination. Patients begin with their arm at rest and gradually abduct their arm to maximum elevation (**B-D**).

The DDR Advanced U-Arm System (Konica Minolta, Wayne, NJ, USA) was used to image and analyze all shoulders in this study (Fig. 1*A*). DDR captures a series of pulsed radiographs during shoulder elevation motion at 6-15 Hz for up to 20 seconds. The entrance surface dose of radiation exposure for an average DDR shoulder series is 1.33 mGy, approximately 1.3x a standard 2-view shoulder static radiograph. The anteroposterior oblique Grashey view was utilized for the optimal view, perpendicular to the plane of GH motion. Licensed radiology technicians trained in the standard DDR protocol conducted the imaging. Patients were both instructed verbally and shown a video on the appropriate tempo of arm elevation during imaging acquisition. To standardize motion and initial position of the scapula, all patients were upright and remained standing as they were directed to start with their arm at rest and move to maximal elevation while supinating the forearm (Fig. 1*B*-*D*; Video 1).

### Dynamic digital radiography reliability measurements

**Figure 2:**
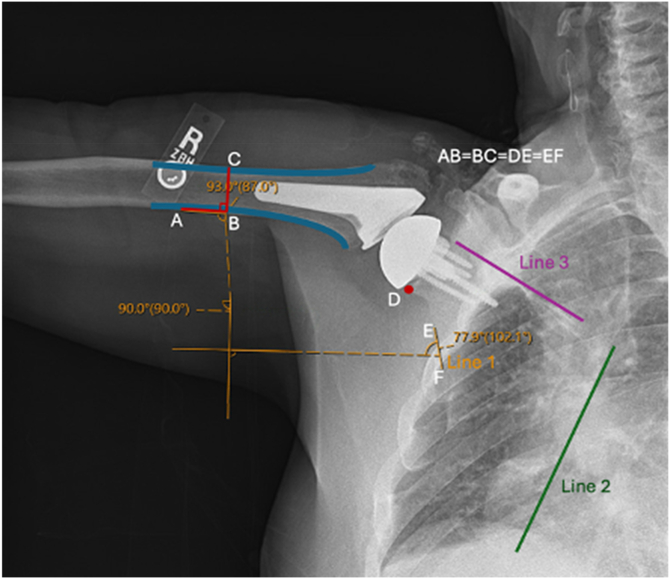
Measurement technique of SHR. The vertical and horizontal reference lines were drawn to over the image. Glenohumeral angles were measured by the angle subtended by the vertical reference line and a line down the medial cortex of the humerus *Line AB*. Scapulothoracic angles were measured by the angle subtended by the horizontal reference line and a line parallel to the lateral border of the scapula *Line EF*. Lines AB and EF are equal in distance and based on the diameter of the humeral shaft *Line CB*. *SHR*, scapulohumeral rhythm.

**Figure 3:**
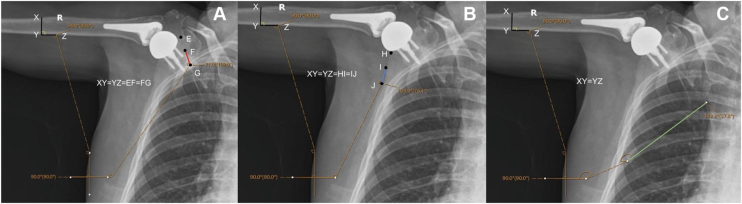
Various measurement techniques for the scapulothoracic angle. The scapulothoracic angle was measured by the angle subtended by the horizontal reference line and either a line parallel to the scapular spine (**A**), lateral border of the scapula (**B**), or medial border of the scapula (**C**).

To assess the reliability of various bony landmarks for manual measurements, Picture Archiving and Communication System software (Sectra Medical, Linköping, Sweden) was used to quantify GH and ST angles manually on each radiograph. Measurements were performed by 2 authors on the same DDR frames at every 10° of shoulder elevation from rest to 120°. DDR frames closest to the interval endpoints, defined as within 5 degrees, were used for measurements. Vertical and horizontal reference lines were drawn first to overlay each image. The GH angle was recorded as the angle subtended by the vertical reference line and a line drawn down the medial cortex of the humerus (Fig. 2). Three possible bony landmarks to measure the ST angle were identified, including the lateral border of the scapula, medial border of the scapula, and scapula spine. The ST angle was calculated using these 3 different techniques in order to determine which was the most reliable. ST angles were calculated by the angle subtended by the horizontal reference line and a line parallel to either A) the lateral border of the scapula, B) the medial border of the scapula, or C) the scapula spine (Fig. 3).

### Dynamic digital radiography validation measurements

GH and ST angle measurements performed at rest, 30°, 60°, 90°, and 120° were used to calculate SHR at several intervals of shoulder elevation. Following interclass correlation analysis to calculate the inter-rater reliability of all ST angle measurement techniques, ST angles using the lateral border of the scapula were used for SHR calculations. SHR was calculated by dividing the change in GH elevation (ΔGH) by the change in ST upward elevation (ΔST)—using the formula SHR = ΔGH/ΔST—between different intervals of shoulder elevation. SHR was calculated across the total range of motion (ROM) from rest to 120°, as well as the rest-30°, 30-60°, 60-90°, and 90-120° elevation intervals.

### Three-dimensional to 2-dimensional model-image registration technique

**Figure 4:**
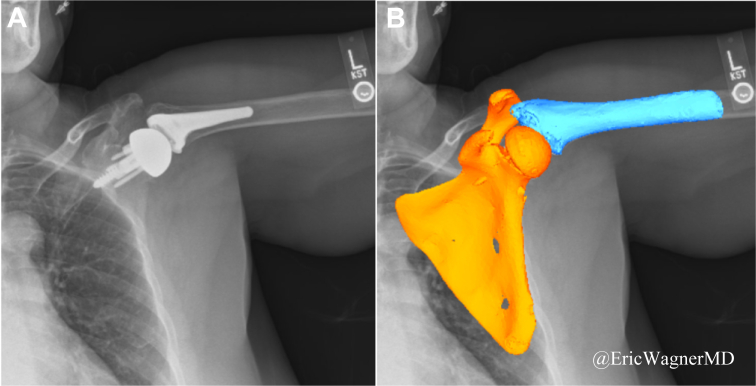
Example of 3D position and orientation of the scapula and humerus models determined using model-image registration technique in JointTrack (University of Florida, Gainesville, FL, USA) and projected onto a dynamic digital radiography (DDR) image; these 3D poses were iteratively adjusted to match their silhouettes with the silhouettes in the DDR image (**A**) shows the DDR image, while (**B**) shows the model image projected. *3D*, 3-dimensional.

Using CT images, 3D surface models of the scapula and humerus pre-operatively and post-operatively with implants were generated with segmentation software (ITK-SNAP, Penn Image Computing and Science Laboratory, Philadelphia, PA, USA) (Fig. 4).^21^^,^^23^^,^^35^^,^^36^ When comparing CT-derived models to the corresponding computer-aided design models, a strong modeling accuracy was previously confirmed by deviation analysis, with a root mean square error of 0.4 mm.^43^ The pre-operative and post-operative scapula and humerus models were overlaid to align the x-, y-, and z-axes of the anatomic coordinate system consistently.^34^^,^^36^ The x-, y-, and z-axes of all models were set in the mediolateral, superoinferior, and anteroposterior directions, respectively. In order to account for glenoid deformities, the pre-operative and post-operative scapular x-axes were aligned with the modified Friedman's line.^11^ The pre-operative scapular origin was defined as the midpoint of the line connecting the most superior and inferior bony edges of the glenoid, while the post-operative scapular origin was set as the center of the glenosphere.^34^^,^^35^ The pre-operative humeral origin was set at the center of the humeral head, and the post-operative humeral origin was defined at the center of the glenosphere.^34^^,^^35^

Using a model-image registration technique in an open-source software program (JointTrack, University of Florida, Gainesville, FL, USA),^1^^,^^33^ 3D position and orientation models of the scapula and humerus were created. These models were then projected onto DDR images, and their 3D poses were adjusted iteratively to match the silhouette of each image. The accuracy of this matching method has been found to be 0.5 mm and 0.8° for in-plane motions.^23^

Cardan angles (x-y-z), which aid in modeling rotational movement, were used to determine humeral and scapular kinematics relative to the x-ray coordinate system, as well as the kinematics of the humerus relative to the scapula.^32^ Internal and external rotation were defined as rotation about the humeral y-axis, and humeral elevation was defined as rotation about the humeral z-axis. Scapular kinematics were defined as anterior/posterior tilt about the x-axis, internal/external rotation about the y-axis, and upward/downward rotation about the z-axis.

The GH and ST parameters were calculated at 10° increments from rest to 120° of shoulder elevation. SHR was calculated as previously described for total ROM below 120° and for the following intervals: rest-30°, 30-60°, 60-90°, and 90-120° of shoulder elevation.

### Statistical analysis

Descriptive statistics were used to summarize the data, including percentages and counts for categorical and ordinal data. Interclass correlation and intraclass correlation (ICC) analyses were used to calculate the inter-rater reliability between the 2 measurers for all GH and ST angles based on the previously described bony landmarks. To assess the validity between our manual measurement technique on DDR, using the medial cortex of the humerus and the lateral border of the scapula, and the 3D to 2D model registration technique (gold standard), paired 2-tailed *t*-tests were conducted. Reliability was assessed using a two-way random effects reliability with absolute agreement, ICC (2, k). Additionally, Bland-Altman plots were used to analyze the 2 measurement techniques. All analyses were carried out using IBM SPSS Statistical Analysis Software (v 29.0; IBM Corp., Armonk, NY, USA). A *P* value of less than 0.05 was considered statistically significant.

## Results

### Patient demographics

A total of 22 shoulders from 21 patients who underwent rTSA and who had both pre-operative and post-operative CT imaging as well as DDR imaging at least 6 months post-operative were included. Patient demographics and other summary characteristics can be found in Table I. Pre-operative diagnoses include 13 shoulders with GH osteoarthritis and 9 shoulders with massive irreparable rotator cuff tears or rotator cuff arthropathy. An inlay humeral prosthesis with a 145° neck shaft angle combined with a lateralized glenoid using either metallic (n = 15) or bony (n = 2) lateralization was used on 17 shoulders. Five shoulders used an onlay humeral prosthesis with a 135° neck shaft angle and a lateralized glenoid using either metallic (n = 4) or bony (n = 1) lateralization.

**Table I:** Demographics data.

Variables	rTSA (n = 22)
Age, mean ± SD (yr)	69.5 ± 10.3
Sex-female, n (%)	16 (72.7%)
Laterality-right, n (%)	11 (50.0%)
Smoking status, n (%)	
Current	1 (4.5%)
Former	4 (18.2%)
Diabetes mellitus, n (%)	1 (4.5%)
Time from surgery to DDR acquisition, mean ± SD (mo)	13.2 ± 9.6

*rTSA*, reverse shoulder arthroplasty; *DDR*, dynamic digital radiography; *SD*, standard deviation.

### Glenohumeral and scapulothoracic angle inter-rater and intrarater reliabilities

Each measurer recorded 105 GH angles and 315 ST angles (105 using the lateral border of the scapula, 105 using the medial border of the scapula, and 105 using the scapula spine). An interclass correlation coefficient statistic was calculated for each variable of interest between the 2 measurers. The interclass correlation coefficient for all GH angles was 0.989 (*P* < .001). The interclass correlation coefficient for ST angles using the lateral border of the scapula was 0.955 (*P* < .001). In contrast, the interclass correlation coefficients for ST angles using the medial border of the scapula and scapula spine were 0.544 (*P* = .101) and 0.142 (*P* = .412), respectively. Additionally, the ICCs demonstrated statistically significant reliability for the GH angles (0.999 (*P* < .001) and for all ST angle measurements using the lateral border, medial border, and scapular spine (0.999, 0.963, and 0.989, respectively; all *P* < .001).

### Manual vs. 3-dimensional to 2-dimensional measurements

**Figure 5:**
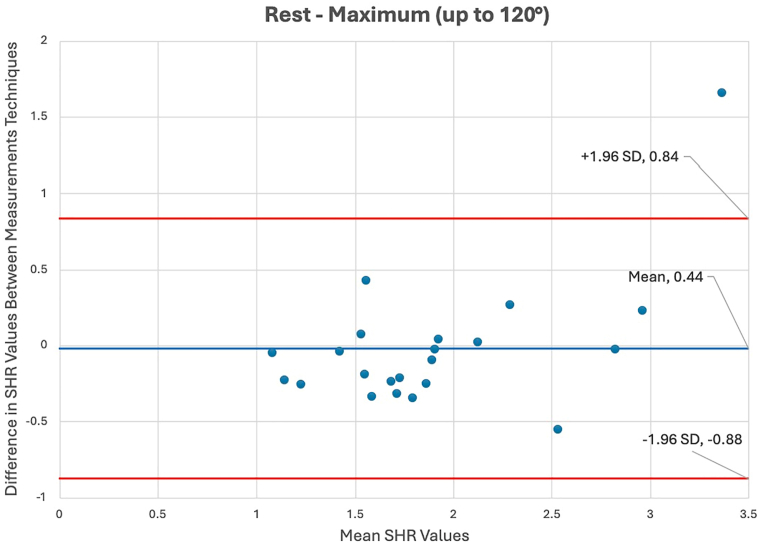
Bland-Altman plot comparing scapulohumeral rhythm (SHR) from rest to maximum (up to 120°) between the manual dynamic digital radiography and 3D to 2D registration measurement techniques. *3D*, 3-dimensional; *2D*, 2-dimensional; *SHR*, scapulohumeral rhythm; *SD*, standard deviation.

Paired 2-tailed *t*-tests revealed no significant differences in SHR values across all intervals of shoulder elevation, indicating strong consistency between the 2 modalities (Table II). The mean total SHRs from rest to 120° for the 3D to 2D registration and manual techniques were 1.91 and 1.88 (*P* = .82), respectively. The interclass correlation between manual and 3D to 2D registration techniques was found to be 0.764 (*P* < .001) for total ROM (Table III). Additionally, Bland-Altman plots demonstrated strong agreement between the 2 measurement techniques (Fig. 5).

**Table II:** Scapulohumeral rhythm measured via 3D-2D registration and manual methods by shoulder abduction interval.

Intervals of glenohumeral abduction	3D-2D registration	Manual	*P* value
Rest-maximum (up to 120°)	1.91 ± 0.73	1.88 ± 0.50	.823
Rest-30°	2.97 ± 1.45	2.47 ± 2.02	.236
30°-60°	1.95 ± 0.56	1.92 ± 0.94	.898
60°-90°	1.92 ± 0.82	1.87 ± 0.66	.673
90°-120°	1.90 ± 0.51	2.24 ± 0.76	.256

*3D*, 3-dimensional; *2D*, 2-dimensional.

Values reported as mean ± standard deviation.

**Table III:** Intraclass correlation values between 3D-2D registration and manual methods by shoulder abduction interval.

Intervals of shoulder abduction	ICC	Quality of agreement	*P* value
Rest-maximum (up to 120°)	0.764	Good-excellent	**<.001**
Rest-30°	0.627	Moderate	**.005**
30°-60°	0.538	Moderate	**.007**
60°-90°	0.821	Good-excellent	**<.001**
90°-120°	−0.026	Poor	.534

*3D*, 3-dimensional; *2D*, 2-dimensional; *ICC*, intraclass correlation.

Bold *P* value denotes statistical significance.

## Discussion

ST motion represents a major component of overall shoulder motion, yet it remains poorly understood. It naturally is a large part of the patient's ultimate post-operative ROM and clinical outcomes.^20^^,^^28^^,^^40^ In particular, increased scapular internal rotation and anterior tilt have been associated with worse shoulder flexion, abduction, shoulder pain and disability index scores, and pain and higher complication rates.^40^ Furthermore, higher post-operative SHRs have been correlated with improved patient-reported outcome measures after shoulder arthroplasty.^20^ Numerous techniques have been used to quantify scapular rotation and associated SHR, including static plane film x-rays,^10^^,^^12^^,^^18^ biplanar fluoroscopy,^13^^,^^14^^,^^26^ goniometry,^42^ skin or electromagnetic sensors,^2^^,^^4^ and palpation techniques.^47^ However, all have issues with reliability, radiation dose, and implementation into a clinical workflow. Finally, 3D registration technology is considered a highly accurate modality for assessing SHR but is resource-consuming and more applicable for a laboratory setting.^13^^,^^36^ DDR offers a promising in-office modality capable of dynamically capturing the GH and ST contributions to shoulder motion perpendicular to the scapular plane of rotation with comparable (1.3x) radiation exposure to a static plain film radiograph. However, a validation of its reliability and accuracy is critical for wider spread adoption.

Ultimately, when assessing the most reliable method to measure SHR using DDR, we found that the medial cortex of the humerus to be an extremely reliable bony landmark for measuring the GH angle and the lateral border of the scapula to be the most reliable bony landmark for measuring the ST angle. Furthermore, SHR measurements calculated manually from 2D DDR frames were not significantly different to those derived from the 3D to 2D registration technique across all intervals of shoulder elevation, indicating technique validity in the plane of the scapula.

Radiographically measuring SHR in standardized imaging software on the Grashey view requires finding a straight segment of humerus subtended from a fixed segment (GH angle) and a straight segment of scapula subtended from a fixed segment (ST angle). Finding these straight osseous segments that have minimal out-of-plane motion during elevation is important to reliably estimate these angles. Additionally, it is important to use a landmark that could be used after implantation of a glenoid prosthesis and would not be altered by surgery. A straight segment of bone is reliably found one cortical diameter distal to the surgical neck on the outer medial surface of the medial humerus. Among potential options for an osseous segment on the scapula, the lateral border was found to be the most reliably visible bony landmark. This thicker segment of bone makes it more radiopaque and is not concealed by the thorax, unlike the medial border. Khawaja et al first used the lateral border of the scapula to quantify SHR in both the diagnosis of scapular pathologies and the assessment of post-operative outcomes and restoration of shoulder biomechanics.^25^ They observed a decreased ST contribution and a corresponding elevated total SHR (up to 90°) of 7.8:1 when the scapula was partially paralyzed from serratus anterior paralysis. Following a split pectoralis major transfer to reconstruct the serratus anterior, the scapular contribution increased, lowering the total SHR to 2.7:1, within range of a healthy physiologic shoulder.^4^^,^^13^ Consistent visibility of the lateral border of the scapula as a landmark is especially important when considering future tracking with computer vision for automated measurements. In contrast, Xiao et al utilized the medial border of the scapula to measure ST angles of various pathological and healthy shoulders, proposing that the total ROM SHR (up to 90°) of normal shoulders may be closer to 3.5:1.^48^ However, using the medial border of the scapula may overestimate SHR.^25^ While use of the lateral border may theoretically underestimate SHR, it lies parallel to the scapular plane and relatively perpendicular to the axis of scapular external rotation, thereby minimizing the magnitude of any potentiation underestimation of motion. Furthermore, the medial border of the scapula is often occluded by the thorax, limiting its utility—supported by a lower ICC in our study. Similarly, using the upper cortical border of the scapular spine to measure ST angles results in a lower ICC, likely due to its out-of-plane motion with scapular tilt (posterior/anterior) and rotation (internal/external) during shoulder elevation^3^^,^^39^ which cannot be accurately captured by 2D radiographs.

3D to 2D registration analyses remain an established modality for assessing in vivo shoulder biomechanics. Studies have demonstrated its high accuracy, comparable to other advanced methods such as biplanar fluoroscopy.^19^^,^^35^ In addition to SHR, these techniques can provide detailed insights into superior/inferior and external/internal translation of the GH joint,^35^ acromial impingement,^21^ and implant balance/positioning.^19^ By matching the 3D bone model to fluoroscopic images, this method enables dynamic and independent assessments of the humerus and scapula across multiple planes of motion. Among these, the scapular plane is most important, as it most closely mirrors natural shoulder movement patterns and biomechanics, optimizing joint congruency while minimizing impingement risks.^35^^,^^38^^,^^42^ Matsuki et al employed 3D to 2D model registration to highlight the plane of elevation's role in preserving joint stability and preventing impingement.^35^ Additionally, by decomposing 3D motion into orthogonal planes, 3D to 2D techniques can identify and quantify shoulder elevation in planes other than the scapular plane. Although 2D DDR is unable to assess multiple planes of motion, it can still provide valuable information on shoulder biomechanics, particularly following total shoulder arthroplasty, in the scapular plane. Our study found this information to be similar in accuracy to that obtained from the 3D techniques but acquired and analyzed in significantly less time, making it more practical in a clinical setting. While 3D to 2D registration technique required roughly two to three days of analysis per shoulder to complete in an independent research laboratory, DDR is integrated into the clinical workflow at our institution and takes no longer than a standard static 2-view shoulder radiographic exam. Manual DDR measurement can be completed in two to five minutes, depending on the measurer experience.

The scapula is known to markedly compensate for the arthritic and postarthroplasty shoulder by providing a major contribution towards shoulder elevation in the form of ST motion via scapula upward rotation, generating a lower SHR.^9^^,^^15^^,^^16^^,^^27^^,^^29^^,^^30^ Indeed, a meta-analysis found 48 rTSA shoulders to have an SHR roughly 1.2 “points” lower than 63 healthy control shoulders.^15^ This has also been demonstrated in several studies using DDR.^16^^,^^22^^,^^45^^,^^48^ Hussain et al found the SHR of 30 rTSA shoulders to be 1.83:1,^16^ aligning closely with our findings of 1.88:1 and 1.91:1 using manual DDR measurements and 3D to 2D registration, respectively. These results have been corroborated by multiple studies on similar patient populations.^8^^,^^19^, ^20^, ^21^^,^^23^^,^^34^^,^^36^

Although additional studies are needed to further establish the utility of DDR diagnosing shoulder pathologies and monitoring improvements following treatment, this study demonstrates the accuracy and reliability of this novel technique assessing shoulder kinematics perpendicular to the plane of scapular rotation and provides a foundation for future studies. 3D to 2D techniques provide substantially more data for research purposes, but the resource-intensive measurement process makes it only practical in a research setting. In vivo shoulder kinematic analysis using DDR may bridge the gap by aiming to acquire the most clinically useful information in a practical and efficient manner. DDR provides a low-radiation, clinic-based method for capturing in vivo biomechanics that allows for rapid SHR analysis.^5^ In comparison to biplanar fluoroscopy, which exposes individuals to increased radiation and takes up to 30 minutes to complete in an external laboratory,^14^ DDR has a comparable level of radiation exposure and time to complete as a standard static 2-view shoulder radiographic exam, enabling it to be performed in an outpatient clinic setting, and thus both a time and cost-effective option that can be scaled for widespread clinical usage. For a patient in the clinic, reliable SHR quantification in the scapular plane can provide diagnostic value, especially for disease processes that have dynamic components, guide surgical planning, and enable pre-operative to post-operative comparisons.^16^^,^^24^^,^^25^ Validation and reliability of the SHR measurement techniques enable further valid use of this technique for many other shoulder pathologies.^16^^,^^17^^,^^25^ Thus, this study serves as a foundation for future work to build upon these findings to better assess shoulder kinematics and define its role as a diagnostic tool, clinical monitoring modality, and determinant of patient outcomes.

This study does have several limitations. Firstly, this study solely serves as a validation and reliability study; therefore, future studies are required to further establish the utility of DDR. We used a sample of rTSA patients for ease of measuring scapula motion given the radiopaque baseplate was fixed to the scapula. Uninstrumented scapula axes have been reported and could have been used instead, albeit with more resources.^23^ Although we used a population of rTSA patients, we believe the measurement technique can be extrapolated to validate SHR measurements in non-instrumented shoulders. However, in conditions where there is substantial clinically significant out-of-plane motion, for example, in ST abnormal motion, this validation may not be accurate. Additionally, the measures assume no thoracic motion or tilt during arm elevation, as measures were made relative to vertical and horizontal lines and not to reference lines in the bony thorax. Out-of-plane motion cannot be captured with this technique, though this does not substantially impair the ability to evaluate the scapular plane. Finally, the utilization of DDR requires specialist equipment that cannot be performed with a standard X-ray machine.

Subsequent studies will further explore the relationship between SHR metrics and patient-reported outcome measures,^20^ reinforcing the utility of in vivo SHR analysis in monitoring post-operative recovery. Establishing a framework for measuring SHR enables the development of automated measurements using computer vision, which would further enhance the technology's scalability. Additionally, once automated using machine learning or artificial intelligence, reliance on a single straight segment on each bone may be obviated if multiple points can be used to generate superior accuracy. Finally, mapping dynamic scapula motion and SHR may eventually play an important role in planning shoulder procedures, such as shoulder arthroplasty, with optimized impingement-free ROM that actually incorporates the scapula and, thus, mimics reality.

## Conclusion

The clinical relevance of shoulder biomechanics remains a complex topic to assess effectively. Our study found the medial border of the humerus and the lateral border of the scapula to be extremely reliable bony landmarks to measure the GH and ST angles in calculating SHR and ultimately understanding shoulder kinematics. DDR provides a novel imaging modality to analyze SHR in the scapular plane of rotation and demonstrates comparable accuracy and validity to that of laboratory-restricted and time-consuming 3D imaging modalities. Resource-light SHR analysis could aid in diagnosis and monitoring response to intervention in many shoulder pathologies.

## Disclaimers:

Funding: No funding was disclosed by the authors.

Conflicts of interest: Eric R. Wagner is a consultant for Stryker, Smith & Nephew, DePuy Synthes, and Acumed and receives institutional research support from Konica Minolta. Michael B. Gottschalk receives institutional support from Skeletal Dynamics, Acumed, and Arthrex and received research support from Stryker and Konica Minolta. Scott A. Banks is a consultant and receives royalties from Enovis and Stryker. Thomas W. Wright is a consultant and receives royalties from Exactech Inc; and is a consultant for ABYRX. Any additional authors, their immediate families, and any research foundations with which they are affiliated have not received any financial payments or other benefits from any commercial entity related to the subject of this article.

## Supplementary Data

Video 1 Dynamic digital radiography of a reverse shoulder arthroplasty shoulde.
